# Supplementary material for: Conservation and divergence of canonical and non-canonical imprinting in murids
Source: Genome Biol. 2023 Mar 14;24:48. doi: 10.1186/s13059-023-02869-1 (PMC10012579; doi:10.1186/s13059-023-02869-1)
Supplement: Supplementary file 10 — Additional file 10. Review history. [file 13059_2023_2869_MOESM10_ESM.docx]

Review History

**First round of review**

**Reviewer 1**

**Were you able to assess all statistics in the manuscript, including the appropriateness of statistical tests used?**

No, I do not feel adequately qualified to assess the statistics.

**Comments to author:**

This study represents an unbiased analysis of genomic imprinting in the rat. Its premise is that only 13 imprinted genes have been identified in rat, and all of these have been identified from known imprinted genes in mouse or human. Twelve of the 13 fall into the 'canonical' imprinted category, i.e., those dependent on gametic differences in DNA methylation, so previous knowledge largely excludes the relatively newly described 'non-canonical' mode of imprinting, which is based on repressive chromatin (H3K27me3) states in oocytes and for which monoallelic expression becomes confined to placental lineages. Systematic and unbiased identification of imprinted genes in the rat would be beneficial for the understanding of the evolution of imprinting and imprinting mechanisms, and because the rat is regarded as superior to the mouse as rodent model for some physiological studies.

The study uses a robust and classic experimental design, using reciprocal crosses amongst three rat strains to enable allele-specific analysis of RNA-seq and WGBS datasets generated from embryo (epiblast) and extra-embryonic (ectoplacental cone, EPC) portions of e8.5 F1 embryos. Candidate imprinted loci are then analysed with respect to existing sperm and oocyte WGBS datasets from the authors, to infer canonical or non-canonical status. This analysis is augmented by new CUT&RUN datasets for H3K27me3 (importantly), H3K4me3 and H3K36me3, as well as accessing published H3K27me3 datasets for rat preimplantation embryos (Lu et al. 2021).

Although there are many existing imprinting datasets, the authors performed comparable RNA-seq and WGBS in mouse at e7.5 in order to investigate the conservation of imprinted genes identified in rat. This led to the identification of eight rat-specific imprinted genes, some of which have properties consistent with non-canonical imprinting. Further analyses of these loci reveal a number of mechanisms by which species-specific imprinting emerges, such as differences in oocyte H3K27me3 domains (which can differ quite subtly), potentially the timing of H3K27me3 reprogramming in preimplantation embryos (which is challenging to quantify), emergence of ZFP57 binding motifs instrumental in allelic methylation maintenance, and endogenous retroviral promoter insertions. The latter extends prior examples of the role of LTRs in the evolution of imprinting via DNA methylation domains in oocytes (Bogutz et al. 2019).

The manuscript also includes a section comparing chromatin profiles of rat and mouse oocytes, and a final section evaluating X-chromosome inactivation status in rat epiblast and EPC, as inferred from allelic RNA-seq analysis.

This is a well-conducted study that leads to some important inferences about the evolution of imprinting mechanisms, revealing an unexpected divergence even within muridae. This may be evidence of rapid evolution, especially amongst imprinted genes with placental functions/roles in fetal growth regulation, and because of the implication of remnants of retroviral promoter insertions in particular in the non-canonical mode of imprinting (Hanna et al. 2019). It will be of considerable interest to the imprinting community.

Specific comments:

1. Although the authors state, lines 521-523: "Autosomal transcripts are mostly biallelically expressed in epiblasts and EPCs, indicating that maternal decidua contamination is minimal", they also record that (lines 334-336 and Figure 1 legend): "33 genes that were scored as maternally expressed in rat EPCs were also found to be highly expressed (RPKM>1 from either allele) in adult blood." These genes are not followed up in their subsequent analysis, leading to the conclusion that they regard them as insignificant. Alternatively, they could represent hits arising from maternal cell contamination in the EPC samples which, in our experience, is difficult to exclude. The authors need to state this as a possibility.

2. Lenience in DMR identification: the authors identify >45,000 DMRs in EPC, but from inspection of Supplementary Table 2, most of these represent not more than a handful of contiguous CpGs. The authors characterise their analysis as 'agnostic', and they then filter on the basis of gametic methylation differences, etc., to arrive at 45 DMRs that were shared in all samples, which include gDMRs and secondary DMRs of many conserved imprinted loci. But the lenience of the analysis must give rise to a huge rate of false discovery, especially since the inclusion criteria required CpGs to be covered by as few as 1 maternal or paternal reads per sample (line 1031). The authors need to provide greater caveats in the text.

Here, again, the greater number of DMRs identified in EPC and the excess of DMRs with maternal-allele methylation could suggest a degree of maternal cell contamination, especially given the lenient threshold (>10% methylation difference). The possibility of maternal cell contamination should be stated.

3. Conservation of imprinting: the authors identify 21 genes with conserved imprinted status between mouse and rat after interrogating their mouse RNA-seq and WGBS datasets. This seems to be an unexpectedly low number; for example, using a similar experimental design, Hanna et al. 2019 detected >50 imprinted loci in mouse extra-embryonic tissues, and Andergassen et al. 2017 detected >40. Based on this apparent shortfall in the number identified in the current work, it would be prudent for the authors to state that their analysis may not be exhaustive and the rat 'imprintome' may include additional genes (even leaving aside tissue-specific effects).

4. Figure 6b: from the violin plots particularly of the mouse reciprocal crosses, there seems to be a skew in allelic distributions (comparing CJ and JC), which might indicate a slight mapping bias, for example if SNP annotations for the J strain are incomplete. Or do the authors interpret this as skewed XCI? Some clarification in the figure legend would be useful.

5. It might be useful to have a summary table of the 8 novel rat-specific imprinted loci. This does exist in part in Figure 3b, but a simplified table/figure not buried in a complex multi-panel would help bring the analysis together.

**Reviewer 2**

**Were you able to assess all statistics in the manuscript, including the appropriateness of statistical tests used?**

No, I do not feel adequately qualified to assess the statistics.

**Comments to author:**

The manuscript entitled "Conservation and divergence of canonical and non-canonical imprinting in murids" by Albert et al. reported their results on comprehensive analyses of rat epiblast (Epi) and ectoplacental cone (EPC) using RNAseq and WGBS on reciprocal F1 hybrid rat and compared with those of mice. They identified 30 imprinted genes in rat, including both canonical and non-canonical imprinted genes. Among them, they demonstrated 8 rat-specific non-canonical imprinting genes. This is an important finding that mouse and rat have different non-canonical imprinted genes in EPC, indicating evolution of non-canonical imprinting among murid. They discuss a variety of mechanism that caused these changes between mouse and rat. However, compared to non-canonical imprinted genes, their analysis on the canonical imprinting is incomplete because the number of imprinted gene identified is too small compared with total number of imprinted genes in mouse. Therefore, the readers cannot understand how much the canonical imprinting is conserved in rat. In addition, the manuscript is hard to read, it is difficult to understand even the number of imprinted genes and DMRs. If the authors would like to insist their conclusion that this work provides a comprehensive list of imprinted loci in the rat and reveal the extent of conservation of imprinted gene expression, it is necessary for them to address several issues as below.

Major points

1. The authors should provide a full picture of this study just in two tables, one containing the information of 1) the numbers of canonical and non-canonical imprinted genes, 2) their paternally or maternally expression, 3) the conservation in humans and other eutherian species, such as cow and pig, 4) rodent- specific or rat-specific genes and 6) mouse-specific genes, if any, and another containing the conservation of canonical imprinted gene clusters including the list of imprinted genes there.

2. Fig. 1d: Peg10 and Sgce are classified in different categories, but this is misleading because they are likely regulated by the same DMR. Therefore, the imprinted genes in the Fig. 1d should be classified 1) maternally and 2) paternally expressed canonical imprinted genes, and paternally expressed non-canonical imprinted genes. I think it fits better the concept of this manuscript.

3. Even the comprehensive analyses on rat Epi and EPC samples, the number of imprinted genes identified is too small, only 30 imprinted genes compare to nearly 200 mouse imprinted genes although it showed much improvement compared with the previous number of 13. Is it the limitation of this study using rat Epi and EPC samples? Authors should present the numbers of imprinted genes and DMR regions in the mouse study they carried out as control experiment.

4. In lines 157-165, the authors said that "In total, 19 paternally and 11 maternally expressed imprinted genes were identified in rat epiblast and EPCs using stringent cutoffs defined above. To identify genes that show a bias in parental expression levels (in addition to monoallelic expression), we performed linear modeling of allele specific data using Limma. An additional 8 paternally and 8 maternally expressed genes were identified using conservative cutoffs."

Then, which are the genes corresponding to the 19 paternally and 11 maternally expressed imprinted genes and the additional 8 paternally and 8 maternally expressed genes in Fig. 1d? Maybe, they correspond to the genes listed in Sup table 1, but it is not easy to understand. Which is the total number of imprinted genes in this work, 30 or 46 (30+8+8)? In addition, the name of Ube3d, Arhgap32, Tmem30b, Tmem30b, Wdr27, Aig1 and Pomt1 in Fig. 1d did not appear in the text. Therefore, they should present the gene names in the text because it is very hard to understand the content of the Sup table 1. The authors should also comment on these genes whether they are associated with DMRs in the text.

5. Paternally expressed Rtl1, Dlk1 and Dio3 are not identified in this study although maternally expressed Rtl1AS, Meg3 and miRNA clusters in the same imprinted gene region are detected and shown in the figures and tables. Is it the limitation of this study mentioned in the comment 3.

6. Related to comment 1, the important feature of canonical imprinted regions is that they form cluster of multiple imprinted genes. Although they presented some examples of H19/Igf2, Trpm5/Tssc4/Alsc2/Cd81 and Peg10/Sgce in line 172, however, other imprinted genes are also included in these imprinted regions, such as Igf2as, Ins2 in the H19/Igf2 region and Dlx5, Calcr, Ppp1r9a, Pon2, Pon3 and Pdk4 in the Peg10 region. How about these genes?

7. In addition, how about the situation of many other well-known imprinted gene clusters, such as Peg3, Nnat, Rtl1, Mest and Ndn imprinted genes because many other genes are included in these imprinted clusters. Without such information, it is not possible to compare the mouse and rat genomic imprinting comprehensively.

8. Line 334: An additional set of 33 genes that were scored as maternally expressed in rat EPCs were also found to be highly expressed (RPKM>1 from either allele) in adult blood (Sup. fig. 4c-d). Are they rat-specific non-canonical imprinted genes? Are they included in the total number of imprinted genes and rat-specific non-canonical imprinted genes or not? If not, why? Is this great difference between mouse and rat a main conclusion of this work?

9. Fig. 1c: What are the reads shown in gray in the Igf2/H19 region? Does it mean that only a small part of Igf2 and H19 transcript exhibit paternal and maternal expression?

10. The authors discussed a variety of mechanism how novel non-canonical imprinted genes emerged. It is better to present them as an additional simple list.

11. Concerning DNA methylation of non-canonical imprinted genes. How do the ERVK LTR in Gab1 methylated? Is the upstream promoter of ERVK in Sall1 methylated? Is H3K27me3 not involved in the case of Gag1? What is the mechanical difference between Gab1 and Sall1?

12. In introduction, the authors introduced that the brown Norway rat is an important model for human pathophysiology and evaluated the utility of the rat in modeling human diseases. In these studies, they clearly demonstrated the difference between rats and humans in terms of no-canonical imprinting. Therefore, this reviewer think that they should comment the applicability and/or limitation of using rat in these studies.

13. What do authors think about the applicability of the variation of the non-canonical imprinted genes to explain species-specific evolution of other eutherian lineages rather than rodents?

14. How do the authors think the difference between rat and mouse contributed to evolution of these species? Is it unique to rodents?

Minor points:

1. Fig. 1d: An asterisk on Sfmbt2 should be omitted because it is not imprinted in human.

2. Supplemental Figure S1a top: It should be Epi, not EB.

3. Line 68: There is an unnecessary space.

4. Figure 2 d: what are the paternally expressed reads in the Kcnq1 region (outside of the box) in the mouse?

5. Figure 3c, d: why the Zfp64 and LOC108358526 reads in EPC are maternally expressed?

**Authors Response**

**Point-by-point responses to the reviewers’ comments:**

**Reviewer #1:**
This study represents an unbiased analysis of genomic imprinting in the rat. Its premise is that only 13 imprinted genes have been identified in rat, and all of these have been identified from known imprinted genes in mouse or human. Twelve of the 13 fall into the 'canonical' imprinted category, i.e., those dependent on gametic differences in DNA methylation, so previous knowledge largely excludes the relatively newly described 'non-canonical' mode of imprinting, which is based on repressive chromatin (H3K27me3) states in oocytes and for which monoallelic expression becomes confined to placental lineages.  Systematic and unbiased identification of imprinted genes in the rat would be beneficial for the understanding of the evolution of imprinting and imprinting mechanisms, and because the rat is regarded as superior to the mouse as rodent model for some physiological studies.

The study uses a robust and classic experimental design, using reciprocal crosses amongst three rat strains to enable allele-specific analysis of RNA-seq and WGBS datasets generated from embryo (epiblast) and extra-embryonic (ectoplacental cone, EPC) portions of e8.5 F1 embryos.  Candidate imprinted loci are then analysed with respect to existing sperm and oocyte WGBS datasets from the authors, to infer canonical or non-canonical status.  This analysis is augmented by new CUT&RUN datasets for H3K27me3 (importantly), H3K4me3 and H3K36me3, as well as accessing published H3K27me3 datasets for rat preimplantation embryos (Lu et al. 2021).

Although there are many existing imprinting datasets, the authors performed comparable RNA-seq and WGBS in mouse at e7.5 in order to investigate the conservation of imprinted genes identified in rat.  This led to the identification of eight rat-specific imprinted genes, some of which have properties consistent with non-canonical imprinting.  Further analyses of these loci reveal a number of mechanisms by which species-specific imprinting emerges, such as differences in oocyte H3K27me3 domains (which can differ quite subtly), potentially the timing of H3K27me3 reprogramming in preimplantation embryos (which is challenging to quantify), emergence of ZFP57 binding motifs instrumental in allelic methylation maintenance, and endogenous retroviral promoter insertions.  The latter extends prior examples of the role of LTRs in the evolution of imprinting via DNA methylation domains in oocytes (Bogutz et al. 2019).
The manuscript also includes a section comparing chromatin profiles of rat and mouse oocytes, and a final section evaluating X-chromosome inactivation status in rat epiblast and EPC, as inferred from allelic RNA-seq analysis.
This is a well-conducted study that leads to some important inferences about the evolution of imprinting mechanisms, revealing an unexpected divergence even within muridae.  This may be evidence of rapid evolution, especially amongst imprinted genes with placental functions/roles in fetal growth regulation, and because of the implication of remnants of retroviral promoter insertions in particular in the non-canonical mode of imprinting (Hanna et al. 2019).  It will be of considerable interest to the imprinting community.

*Reply: We sincerely thank Reviewer #1 for their cogent summary and overall positive review of our study. We address each of their constructive comments and suggestions below.*

Specific comments:
1. Although the authors state, lines 521-523: "Autosomal transcripts are mostly biallelically expressed in epiblasts and EPCs, indicating that maternal decidua contamination is minimal", they also record that (lines 334-336 and Figure 1 legend): "33 genes that were scored as maternally expressed in rat EPCs were also found to be highly expressed (RPKM>1 from either allele) in adult blood."  These genes are not followed up in their subsequent analysis, leading to the conclusion that they regard them as insignificant.  Alternatively, they could represent hits arising from maternal cell contamination in the EPC samples which, in our experience, is difficult to exclude.  The authors need to state this as a possibility.

*Reply: We agree that the 33 genes in question may indeed be imprinted genes, and that we may have been overly stringent in our effort to exclude false-positives. We also agree that it was difficult to exclude such potentially interesting examples of imprinted gene expression. In fact, we had previously generated a version of* ***Figure 1d*** *that represented these (potentially false positive) 33 hits (****Figure R1****).*

**Figure R1**, a previous version of Fig. 1d that includes 33 maternally expressed genes in EPC samples that are also highly expressed in adult blood (dark red).

*Given the fact that the majority of the 33 hits function in blood-related processes (eg. The innate immune response genes Clec4d and Clec4e and leukocyte differentiation gene Spi1, see* ***Table R1*** *for the full list of 33 genes and their known functions), we opted for caution, relegating the 33 hits to a supplemental figure (Sup.* ***Figure S4****) and excluding them from our final list of imprinted genes so that we could focus on the genes that analyzed in greater detail (the conserved canonically expressed imprinted genes and the 8 novel rat non-canonical imprinted genes Gsto1, Rpl39l, Zfp516, Zfp64, Slc38a1, LOC108350526, Syt16AS and Gadl1). We appreciate the reviewer’s comment and again note that it was difficult to choose between presenting* ***Figure 1d*** *or* ***Figure R1 (above)****.*

**Table R1** List of maternally expressed genes in EPC cells only and their associated processes. We include this table in a separate sheet in **Supplemental Table 1.**

| **Gene** | **full name** | **chr** | **Gene ontology (process)** |
| --- | --- | --- | --- |
| Coro1a | coronin, actin binding protein 1A | chr1 | T cell activation |
| Itgal | integrin alpha L | chr1 | cell adhesion/memory T cell extravasation |
| Ifitm1 | interferon induced transmembrane protein 1 | chr1 | response to virus/ immune system process |
| Cd53 | CD53 antigen | chr2 | myoblast fusion |
| Cytip | cytohesin 1 interacting protein | chr3 | regulation of cell adhesion |
| Itga4 | integrin alpha 4 | chr3 | cell-cell adhesion |
| Spi1 | spleen focus forming virus (SFFV) proviral integration oncogene | chr3 | myeloid leukocyte differentiation |
| B2m | beta-2 microglobulin | chr3 | T cell activation |
| Cdc25b | cell division cycle 25B | chr3 | mitotic cell cycle |
| Tubb1 | tubulin, beta 1 class VI | chr3 | hyroid gland development |
| Fgl2 | fibrinogen-like protein 2 | chr4 | memory T cell differentiation |
| Arhgap25 | Rho GTPase activating protein 25 | chr4 | actin filament organization |
| Clec4d | C-type lectin domain family 4, member d | chr4 | antifungal innate immune response |
| Clec4e | C-type lectin domain family 4, member e | chr4 | antifungal innate immune response |
| Zc3h12a | zinc finger CCCH type containing 12A | chr5 | T cell receptor signaling pathway/cellular response to virus |
| Coro1a | coronin, actin binding protein 1A | chr5 | T cell activation/cell migration |
| Pla2g2a | phospholipase A2, group IIA | chr5 | T cell proliferation |
| Timp3 | tissue inhibitor of metalloproteinase 3 | chr7 | mesenchymal cell differentiation involved in bone development |
| Stat6 | signal transducer and activator of transcription 6 | chr7 | response to cytokine |
| Lrg1 | leucine-rich alpha-2-glycoprotein 1 | chr9 | angiogenesis |
| Treml2 | triggering receptor expressed on myeloid cells-like 2 | chr9 | T cell activation |
| Pla2g7 | phospholipase A2, group VII | chr9 | monocyte chemotaxis/inflammatory response |
| Lcp2 | lymphocyte cytosolic protein 2 | chr10 | T cell receptor signaling pathway |
| Dock2 | dedicator of cyto-kinesis 2 | chr10 | cell migration/myoblast fusion/T cell activation |
| Itga2b | integrin alpha 2b | chr10 | cell-cell adhesion/leukocyte migration |
| Mcemp1 | mast cell expressed membrane protein 1 | chr12 | no data |
| Cxcr4 | chemokine (C-X-C motif) receptor 4 | chr13 | immune response/T cell proliferation |
| Ptprc | protein tyrosine phosphatase, receptor type, C | chr13 | T cell receptor signaling pathway |
| F5 | coagulation factor V | chr13 | blood coagulation |
| Plek | pleckstrin | chr14 | actin cytoskeleton reorganization |
| Syk | spleen tyrosine kinase | chr17 | B cell receptor signaling pathway |
| Vim | vimentin | chr17 | intermediate filament organization |
| Cd226 | CD226 antigen | chr18 | T cell receptor signaling pathway |

*The manuscript was revised for clarity as follows:*

*Results section:*

*“While an additional set of 33 genes that were scored as maternally expressed only in rat EPCs, we chose not to study these in greater detail because all were found to be highly expressed (RPKM>1 from either allele) in adult blood and thus may reflect maternal decidua expression (Sup. fig. 5c-d, see Sup. table 1).”*

*Discussion section:*

*“Therefore, the full catalogue imprinting in the rat will require additional studies. […] . In addition, extraembryonic samples are frequently contaminated with maternal decidua, making it difficult to discriminate between maternally expressed imprinted genes in our EPC samples from genes that are normally expressed in adult blood.”*

2. Lenience in DMR identification: the authors identify >45,000 DMRs in EPC, but from inspection of Supplementary Table 2, most of these represent not more than a handful of contiguous CpGs. The authors characterise their analysis as 'agnostic', and they then filter on the basis of gametic methylation differences, etc., to arrive at 45 DMRs that were shared in all samples, which include gDMRs and secondary DMRs of many conserved imprinted loci. But the lenience of the analysis must give rise to a huge rate of false discovery, especially since the inclusion criteria required CpGs to be covered by as few as 1 maternal or paternal reads per sample (line 1031). The authors need to provide greater caveats in the text.

*Reply: We have updated the manuscript to state the caveats that underly the potentially a high rate of false positives caused by our analysis method.*

*Results section:*

*“Notably, while the vast majority of DMRs found in EPCs are maternally methylated (n=40,427, 90%), a roughly equal number of paternal and maternal DMRs were identified in epiblast samples (maternally methylated n=4,386, 48%), which may reflect potential maternal somatic cell contamination or incomplete resetting of parental DNAme levels in EPC samples. To identify imprinted DMRs, we subsequently analyzed our previously generated rat oocyte and sperm WGBS datasets (39) using stringent parameters (39)(see Sup. table 3 for the complete list of* *data generated and analyzed in this study). Integrated analysis of DNAme levels revealed a total of 5,876 regions that show dramatic DNAme differences (delta ≥50%) between gametes and the parental alleles of epiblast or EPCs (Sup. fig. 1b & Sup. table 2).”*

*However, we believe that our filtering criteria (DNAme delta and CpG counts and read counts) are appropriate for allele-specific analyses and that the potentially false positive DMRs are real biological noise. In other words, unwanted biological variability underlies the high number of DMRs identified in EPC samples.*

- *In the revised manuscript, we add that reprogramming of parental DNA methylation levels is incomplete in the placenta (reviewed very recently here: (1)) and that maternal DMRs were also overrepresented in a previous study in human placenta (2). Analysis of our mouse WGBS also uncovered a bias in maternal DMRs in EPC samples, albeit lower than in rat (67% in mouse compared to 89% in rat).*
- *Regardless, another source of biological noise, maternal cell contamination (discussed in more detail below), is pervasive when dissecting extra-embryonic tissues in any mammalian species, and we now state this important caveat in the revised Results and Discussion sections. We note here that unless maternal decidua cells outnumber extra-embryonic cells in our EPC samples, maternally methylated DMRs cannot be present in our second filter for DNAme delta of >50%.*

*Importantly, we do not state that the >45,000 DMRs in rat EPC are imprinted.*

Here, again, the greater number of DMRs identified in EPC and the excess of DMRs with maternal-allele methylation could suggest a degree of maternal cell contamination, especially given the lenient threshold (>10% methylation difference). The possibility of maternal cell contamination should be stated.

*Reply: We agree and have updated the manuscript to include this important caveat.*

3. Conservation of imprinting: the authors identify 21 genes with conserved imprinted status between mouse and rat after interrogating their mouse RNA-seq and WGBS datasets.  This seems to be an unexpectedly low number; for example, using a similar experimental design, Hanna et al. 2019 detected >50 imprinted loci in mouse extra-embryonic tissues, and Andergassen et al. 2017 detected >40.  Based on this apparent shortfall in the number identified in the current work, it would be prudent for the authors to state that their analysis may not be exhaustive and the rat 'imprintome' may include additional genes (even leaving aside tissue-specific effects).

*Reply: We appreciate the reviewer’s insight and agree that the number of shared imprinted genes is not exhaustive. As mentioned, this study cannot identify all rat imprinted genes and we have changed our language from “a comprehensive list of rat imprinted genes” to “expanded” or “agnostic and systematic analysis”.*

*In regards to the number of imprinted genes identified, we note that as a function of measuring conservation of imprinting (how we arrived at the number 21), genes “drop out” from the analysis due to incomplete rat gene annotations and orthologue annotations (see* ***Figure R2****).*

**Figure R2** showing the number of genes from which we can assess allele-specific expression levels in rat (yellow) mouse (green) and both species (red).

*Comparing rat and mouse NCBI gene annotations, the rat (69,194) has 37,326 or 35% fewer transcripts than mouse (106,520) to begin with. We tried minimizing this limitation by conducting de novo transcriptome assembly from our rat RNA-seq data, which added 3,242 transcripts. Nevertheless, the quality of the rat reference is inferior to the mouse, which has benefited from decades of being the most utilized model for mammalian genomics.*

*Our three strain, two reciprocal cross strategy in rat compensated for the relative lack of diversity between rat strains compared to mouse strains. Nevertheless, we only performed RNA-seq on two cell types, precluding the identification of imprinted expression of silenced genes. These limitations to our study are discussed in the revised Discussion section.*

*“However, comprehensive assessment of allele-specific gene expression and DNAme levels depends on naturally occurring genetic variation between parental genomes, which are absent at some regions in the three distinct rat strains used here. Therefore, the full catalogue imprinting in the rat will require additional studies. Further, while we portrayed the embryonic precursor cells to the placenta and the adult soma, some genes are only imprinted in specific adult tissues. In addition, extraembryonic samples are frequently contaminated with maternal decidua, making it difficult to discriminate between maternally expressed imprinted genes in our EPC samples from genes that are normally expressed in adult blood. Finally, the rat reference genome has many incomplete chromosome assemblies and gene model annotations. All of these limitations can be rectified in future studies that profile additional tissues generated from distinct rat strain crosses using an updated reference genome.”*

*We are actually pleased that despite all the shortcomings mentioned above, we nevertheless identified 21 imprinted genes in both rat and mouse, some of which were manually annotated due to a lack of gene model in rat (Kcnq1ot1) or a lack of orthologue annotation (H19). That said, we agree that there are likely additional imprinted genes that we did not capture, and have edited the manuscript to reflect this limitation of the study, including removal of the word “comprehensive” to describe our identified list of imprinted genes.*

4. Figure 6b: from the violin plots particularly of the mouse reciprocal crosses, there seems to be a skew in allelic distributions (comparing CJ and JC), which might indicate a slight mapping bias, for example if SNP annotations for the J strain are incomplete.  Or do the authors interpret this as skewed XCI?  Some clarification in the figure legend would be useful.

*Reply: As noted by the reviewer, the CJ cross (where the paternal genome is JF1) shows less skewed XCI towards the paternal allele compared to the JC cross. Conversely, the JC cross (where C57 is the paternal allele) shows more paternally skewed expression. We indeed suspect that this is due to the inherently lower quality of the JF1 genome, which is based on the high-quality C57 reference genome, despite our pipeline’s attempts to minimize such effects by introducing INDELs to the pseudogenome in addition to SNPs. In other words, we interpret the results from both crosses as skewed XCI, with the JC cross showing a greater degree of XCI. We have added a sentence to clarify this issue in the figure legend as suggested.*

*“Differences in XCI skewing between CJ and JC samples (bottom right) is likely due to differences in genome quality between the parental strains, with reads preferentially aligning to the reference “C” genome.”*

5. It might be useful to have a summary table of the 8 novel rat-specific imprinted loci.  This does exist in part in Figure 3b, but a simplified table/figure not buried in a complex multi-panel would help bring the analysis together.

*Reply: As requested, we include a summary table of the 8 novel rat-specific loci and their salient features. We also include the 3 non-canonically imprinted genes that are conserved in mouse (Sfmbt2, Sall1 and Gab1).*

**Table R2** (**Table 2** in the revised manuscript). Summary of non-canonical imprinted gene expression in the rat.

| **Gene** | **Full name** | **Chr.** | **EPC-DMR (Overlapping LTR-LTR family)** | **Putative mechanism underlying specie-specific imprinting** | **Gene ontology (process)** | **Note** |
| --- | --- | --- | --- | --- | --- | --- |
| *Gsto1* | Glutathione S-Transferase Omega 1 | chr1 | N.I. | unknown | Glutathione metabolic process | |
| *Zfp64* | Zinc finger protein 64 | chr3 | Mat | Oocyte epigenome (DNAme vs H3K27me3) | Mesenchymal cell differentiation | |
| *LOC108350526* | | chr3 | Mat (RMER13A/RLTR20B3-ERVK) | Oocyte epigenome (DNAme vs H3K27me3) | Unknown |  |
| *Syt16AS (LOC102550910)* | Synaptotagmin 16 - antisense | chr6 | Mat (MTC-ERVL) | Unknown | Exocytosis (Syt16) | |
| *Gadl1-3'UTR* | Glutamate Decarboxylase Like 1 | chr8 | Mat (RMER3B-ERVK) | Unknown | Carboxylic acid metabolic process (Gadl1) | |
| *Slc38a1* | Solute Carrier Family 38 Member 1 | chr7 | Mat (RMER6B/ORR1A4/MTD-ERVK/ERVL) | LTR insertion and ZFP57 motifs | Amino acid transmembrane transport | |
| *Rpl39l* | Ribosomal Protein L39 Like | chr10 | Mat | Unknown | Unknown |  |
| *Sfmbt2* | Scm like with four mbt domains 2 | chr17 | Mat | Common imprinting between rats and mice | Negative regulation of gene expression | *Sfmbt2* is essential for trophoblast maintenance and placenta development in mice (3). |
| *Zfp516* | Zinc finger protein 516 | chr18 | Mat | Oocyte epigenome (DNAme vs H3K27me3) | Brown fat cell differentiation | |
| *Sall1* | Spalt Like Transcription Factor 1 | chr19 | Mat (RLTR20B3-ERVK) | Common imprinting between rats and mice | Heart/Kidney/Limb development | |
| *Gab1* | GRB2 Associated Binding Protein 1 | chr19 | Mat (RLTR15-ERVK) | Common imprinting between rats and mice | MAPK cascade/Angiogenesis | *Gab1* is essential for development of placenta (syncytiotrophoblast) in mice. (4). |

*Finally, in response to Reviewer 2 below, we also include a similar table summarizing canonically expressed genes in the rat (****Table R3, Table 1 in the revised manuscript****).*

**Reviewer #2:**

The manuscript entitled "Conservation and divergence of canonical and non-canonical imprinting in murids" by Albert et al. reported their results on comprehensive analyses of rat epiblast (Epi) and ectoplacental cone (EPC) using RNAseq and WGBS on reciprocal F1 hybrid rat and compared with those of mice. They identified 30 imprinted genes in rat, including both canonical and non-canonical imprinted genes. Among them, they demonstrated 8 rat-specific non-canonical imprinting genes. This is an important finding that mouse and rat have different non-canonical imprinted genes in EPC, indicating evolution of non-canonical imprinting among murid.

They discuss a variety of mechanism that caused these changes between mouse and rat. However, compared to non-canonical imprinted genes, their analysis on the canonical imprinting is incomplete because the number of imprinted gene identified is too small compared with total number of imprinted genes in mouse. Therefore, the readers cannot understand how much the canonical imprinting is conserved in rat. In addition, the manuscript is hard to read, it is difficult to understand even the number of imprinted genes and DMRs. If the authors would like to insist their conclusion that this work provides a comprehensive list of imprinted loci in the rat and reveal the extent of conservation of imprinted gene expression, it is necessary for them to address several issues as below.

*Reply: We thank Reviewer #2 for their overall positive outlook on our manuscript. We address all their concerns, including the use of the word “comprehensive”, as outlined in detail below.*

Major points
1.      The authors should provide a full picture of this study just in two tables, one containing the information of 1) the numbers of canonical and non-canonical imprinted genes, 2) their paternally or maternally expression, 3) the conservation in humans and other eutherian species, such as cow and pig, 4) rodent- specific or rat-specific genes and 6) mouse-specific genes, if any, and another containing the conservation of canonical imprinted gene clusters including the list of imprinted genes there.

*Reply: This is a great suggestion. As mentioned above, we now include* ***Table R2****, which summarizes the non-canonically imprinted genes, in the revised manuscript (****Table 2****).*

*Below,* ***Table R3*** *describes the canonically imprinted genes, their genomic location (cluster), the methylated allele in rat and mice and their imprinting conservation.* ***Table R3*** *is included in the revised manuscript (****Table 1****):*

**Table R3** List of canonically imprinted genes identified in the rat and mouse.

| **Genomic loci** | **Chr.** | **gDMR** | **Epi-DMR** | **EPC-DMR** | **Identified imprinted genes** | **DMR type in mice** | **Imprinting in non-rodent mammals**** |
| --- | --- | --- | --- | --- | --- | --- | --- |
| *H19-Igf2* | 1 | Pat | Pat | Pat | *Igf2, H19* | Pat | Human, Cow, Pig, Opossum, Wallaby |
| *Dlk1-Meg3* | 6 | Pat | N.I. | N.I. | *Rtl1AS, Meg3, miRNA cluster* | Pat | Human, Cow, Pig |
| *Rasgrf1* | 8 | Pat | Pat | Pat | *Rasgrf1** | Pat | Pig |
| *Peg3* | 1 | Mat | Mat | Mat | *Peg3** | Mat | Human, Cow, Pig |
| *Snrpn* | 1 | Mat | Mat | Mat | *Snrpn, Snurf, Magel2* | Mat | Human, Cow, Pig |
| *Inpp5f* | 1 | Mat | N.I. | N.I. |  | Mat | Human, Cow, Pig |
| *Kcnq1ot1*† | 1 | Mat | N.I. | N.I. | *Kcnq1ot1, Trpm5, Tssc4, Ascl2, Cd81* | Mat | Human, Cow, Pig |
| *Airn-Igf2r* | 1 | Mat | N.I. | N.I. | *Igf2r** | Mat | Human, Cow, Pig, Opossum, Wallaby |
| *Plagl1* | 1 | Mat | Mat | Mat |  | Mat | Human, Cow, Pig |
| *Mcts2* | 3 | Mat | N.I. | N.I. |  | Mat | Human |
| *Nnat* | 3 | Mat | N.I. | N.I. |  | Mat | Human, Cow, Pig |
| *Gnas (Nespas-GnasXL)* | 3 | Mat | N.I. | N.I. | *Gnas* | Mat | Human, Cow, Pig |
| *Gnas (exon 1A)* | 3 | Mat | Mat | Mat | *Gnas* | Mat | Human |
| *Sgce-Peg10* | 4 | Mat | Mat | Mat | *Sgce, Peg10* | Mat | Human, Cow, Pig, Wallaby |
| *Mest* | 4 | Mat | Mat | Mat |  | Mat | Human, Cow, Opossum, Wallaby |
| *Nap1l5* | 4 | Mat | N.I. | N.I. |  | Mat | Human, Cow, Pig |
| *Trappc9* | 7 | Mat | Mat | Mat |  | Mat | Human |
| *Slc38a4* | 7 | Mat | N.I. | N.I. | *Slc38a4* | Mat | Pig |
| *Zdbf2* | 9 | Mat | N.I. | N.I. | *Zdbf2* | Mat (transient) | Human |
| *Fkbp6* | 12 | Mat | N.I. | N.I. |  | Mat |  |
| *Grb10* | 14 | Mat | Mat | Mat | *Grb10* | Mat | Human |
| *Zrsr1* | 14 | Mat | Mat | Mat | *Zrsr1* | Mat |  |
| *Impact* | 18 | Mat | Mat | Mat | *Impact** | Mat | Cow |
| *Cdh15* | 19 | Mat | N.I. | Mat |  | Mat (transient) |  |

Pat: Paternally methylated, Mat: Maternally methylated, N.I., Not informative

†: Not annotated

*: These genes are reported to be imprinted (expressed) in rats in previous studies (5).

**: One or more genes in the orthologous imprinted cluster have been reported to be imprinted in human, cow (and pig), or marupials (5) (6) (7) (8) (9) (10) (11) (12) (13) (14)

2.      Fig. 1d: Peg10 and Sgce are classified in different categories, but this is misleading because they are likely regulated by the same DMR. Therefore, the imprinted genes in the Fig. 1d should be classified 1) maternally and 2) paternally expressed canonical imprinted genes, and paternally expressed non-canonical imprinted genes. I think it fits better the concept of this manuscript.

*Reply: Indeed, Peg10 and Sgce share an imprinted CpG island promoter in both mouse and rat and we expect both genes to be co-regulated in rat as well (see* ***Figure R3****).*

**Figure R3** conservation of the *Sgce/Peg10* locus. Green lines represent synteny. The known mouse gDMR is shown in red.

*Reply: Closer examination of the underlying allele-specific RNA-seq data showed that Sgce and Peg10 were classified in different categories because Sgce is expressed at insufficient levels in rat epiblast cells to confidently call allele-specific expression, whereas Peg10 was informative in both epiblast and EPC samples (see* ***Figure R4****). As Sgce did not meet our stringent cutoffs in rat epiblast samples, it was categorized as “paternally expressed only in EPC samples”. However, the discrepancy between Sgce expression levels in epiblast and EPC samples is minor, demonstrating that while our cutoffs result in a high confidence list of imprinted genes, such stringency can indeed miss imprinted gene expression. Interestingly, Sgce and Peg10 are expressed >10-fold higher in mouse compared to rat, making imprinted expression in mouse much easier to identify.*

**Figure R4**. *Sgce* and *Peg10* allelic RPM expression values for all samples are shown. Note that *Sgce* expression in rat epiblast samples does not meet our cutoff criteria for calling paternal expression rations (RPM>=0.5). Bar charts correspond to mean allelic RPM values, dots correspond to individual replicates.

*Reply: In other words, the differences in Peg10 and Sgce categorization is simply caused by our expression cutoff values. Given both genes are known to share the same DMR in mouse (as stated by the reviewer), and confirmed in our mouse data (****Fig 2a****), and the synteny of the Peg10/Sgce region between mouse and rat (****Fig. R3****), and the fact that we agnostically identified the rat Sgce/Peg10 gDMR in EPC samples (****Fig. 3a****), Sgce and Peg10 should be categorized together.*

*However, in order to improve the clarity of our manuscript, we simplified* ***Fig. 1d****. Imprinted genes are now categorized as “paternally expressed” or “maternally expressed”, which is consistent with previous karyotype ideograms generated in mouse and human studies. Combined with the new summary tables 1 and 2, we hope this visual representation of our results will be readily accessible to the community.*

3.      Even the comprehensive analyses on rat Epi and EPC samples, the number of imprinted genes identified is too small, only 30 imprinted genes compare to nearly 200 mouse imprinted genes although it showed much improvement compared with the previous number of 13. Is it the limitation of this study using rat Epi and EPC samples?

*Reply: Reviewer 1 (comment #3) raised the same concern. As detailed above, our study does indeed have limitations, which we now discuss in the revised Discussion (see response to Reviewer 1 comment #3):*

*“[…] thereby providing an unprecedented view of the rat imprintome […]. However, comprehensive assessment of allele-specific gene expression and DNAme levels depends on naturally occurring genetic variation between parental genomes, which are absent at some regions in the three distinct rat strains used here. Therefore, the full catalogue imprinting in the rat will require additional studies. Further, while we portrayed the embryonic precursor cells to the placenta and the adult soma, some genes are only imprinted in specific adult tissues. In addition, extraembryonic samples are frequently contaminated with maternal decidua, making it difficult to discriminate between maternally expressed imprinted genes in our EPC samples from genes that are normally expressed in adult blood. Finally, the rat reference genome has many incomplete chromosome assemblies and gene model annotations. All of these limitations can be rectified in future studies that profile additional tissues generated from distinct rat strain crosses using an updated reference genome.”*

*Given these caveats, we softened our wording of “comprehensive analyses” to “genome-wide and systematic analyses” or “extensive” or “unprecedented”. Despite the limitations listed above, of the canonical imprinted genes identified in the rat, all are conserved in the mouse. Further, we nevertheless identified 8 novel imprinted genes in the rat, all of which show evidence of non-canonical imprinting regulation, in line with the rapid evolution of placental imprinting (discussed below).*

Authors should present the numbers of imprinted genes and DMR regions in the mouse study they carried out as control experiment.

*Reply: We have updated* ***Supplemental table 2*** *and generated a new* ***Supplemental Figure 3*** *to describe our findings, including imprinted gene expression and the number of DMRs (452), identified in our mouse control experiments. Briefly, the results are mostly consistent with what we found in rat in terms of number of imprinted genes identified by RNAseq. Given the higher rate of sequence variation between mouse parental strains (1 variant per 120 nucleotides) relative to rat parental strains (1 variant per 622 nucleotides), we not surprisingly identified more DMRs in mouse (452) than rat (45). Notably, we identified 19/20 known mouse gametic DMRs using our mouse epiblast and EPC sample WGBS.*

4.      In lines 157-165, the authors said that "In total, 19 paternally and 11 maternally expressed imprinted genes were identified in rat epiblast and EPCs using stringent cutoffs defined above. To identify genes that show a bias in parental expression levels (in addition to monoallelic expression), we performed linear modeling of allele specific data using Limma. An additional 8 paternally and 8 maternally expressed genes were identified using conservative cutoffs."
Then, which are the genes corresponding to the 19 paternally and 11 maternally expressed imprinted genes and the additional 8 paternally and 8 maternally expressed genes in Fig. 1d?

*Reply: To address the concerns raised, we updated the manuscript and explicitly list the names of the imprinted genes and the method by which they were identified. In the previous text, we meant to describe these 8 paternally and 8 maternally expressed genes as those that were not identified in our first set of statistical tests, so none of the 16 correspond to the 19 paternally and 11 maternally monoallelically expressed genes. We apologize for the lack of clarity.*

*The manuscript has been updated to include the following lists of imprinted genes identified by a stringent T-test:*

*“In total, 18 paternally (Snrpn, Magel2, Peg10, Sgce, Igf2, Zrsr1, Zdbf2, Sfmbt2, Sall1, Gab1, Gsto1, Rpl39l, Zfp516, Zfp64, Slc38a1, LOC108350526, Syt16-AS, Gadl1-3’UTR) and 11* *maternally (Tssc4, Cd81, Ascl2, Trpm5, Mir675, H19, Grb10, Maged2, Meg3, Rtl1-AS, Gnas) expressed imprinted genes were identified”*

*And (additional) genes showing bias in parent-of-origin expression identified by Limma:*

*Additional paternally biased genes identified by Limma: Aig1, Wasf3, Pomt1, Tmem67, Tmem30b, Slc38a4, Arhgap32 and Ube3d.*

*Additional maternally biased genes identified by Limma: Wdr27, Fam196b, Tbx3, Spp1, Igfbp3, LOC103691708 and Cdc42ep1.*

Maybe, they correspond to the genes listed in Sup table 1, but it is not easy to understand.

*Reply: In addition to the explicit lists of gene names included in the revised manuscript (above), we have updated* ***Sup table 1 (sheet 1d)*** *for clarity.*

Which is the total number of imprinted genes in this work, 30 or 46 (30+8+8)? In addition, the name of Ube3d, Arhgap32, Tmem30b, Tmem30b, Wdr27, Aig1 and Pomt1 in Fig. 1d did not appear in the text. Therefore, they should present the gene names in the text because it is very hard to understand the content of the Sup table 1.

*Reply: As addressed above, the total number of rat imprinted genes identified in this work is 30, with 16 biased genes (46 total). We hope this is clearer with the new tables and updated text. As suggested, we now include the full list of gene names in the text.*

The authors should also comment on these genes whether they are associated with DMRs in the text.

*Reply: With the exception of LOC103691708 and Slc38a4, the genes identified using Limma are not associated with DMRs. We have updated the manuscript to include this information:*

*“While genes identified using Limma include the known mouse imprinted gene Slc38a4 and show clear parent-of-origin expression patterns, only Slc38a4 and LOC103691708 are associated with a DMR. We therefore focused subsequent analyses on genes that met our stringent statistical cutoffs, as defined above.”*

5.      Paternally expressed Rtl1, Dlk1 and Dio3 are not identified in this study although maternally expressed Rtl1AS, Meg3 and miRNA clusters in the same imprinted gene region are detected and shown in the figures and tables. Is it the limitation of this study mentioned in the comment 3.

*Reply: As the reviewer noted above, the low genetic variation between inbred rat strains is a limitation of the study (as well as the fewer number of annotated rat genes) that precludes the identification of all known imprinted genes in the rat (see* ***Figure R2*** *above). As discussed above (comment #3), we updated the Discussion section to make this limitation clear, and now refer to our list of rat imprinted genes as “expanded” rather than “comprehensive”.*

*To address the reviewer’s curiosity, here is a summary of our (negative) results for the Meg3 cluster genes listed above:*

- *Rtl1: not expressed in epi or EPC in rat.*
- *Dlk1: moderately expressed in BW and WB epiblast samples, though there are no parental variants over exons. No informative intronic reads were observed either.*
- *Dio3: not expressed in epi or EPC in rat.*

6.      Related to comment 1, the important feature of canonical imprinted regions is that they form cluster of multiple imprinted genes. Although they presented some examples of H19/Igf2, Trpm5/Tssc4/Alsc2/Cd81 and Peg10/Sgce in line 172, however, other imprinted genes are also included in these imprinted regions, such as Igf2as, Ins2 in the H19/Igf2 region and Dlx5, Calcr, Ppp1r9a, Pon2, Pon3 and Pdk4 in the Peg10 region. How about these genes?

*Reply: As addressed above and in the revised manuscript, not all known imprinted genes could be assessed for parent-specific expression, including those in known imprinted clusters, using our experimental design. We briefly summarize our results for the genes mentioned above:*

- *Igf2as (annotated as LOC100909737 in rat): does not overlap parental variants.*
- *Ins2: not expressed in rat epiblast or EPC.*
- *Dlx5: lowly expressed in epi (0.25 RPKM) and EPC (0 RPKM), but some maternal-specific reads are observed in epiblast samples. Dlx5 seems to show imprinted expression, but it’s expression level (allele-agnostic and allele-specific) does not meet our filtering criteria.*
- *Calcr: exons do not overlap any parental variants.*
- *Ppp1r9a: Modestly expressed in epiblast only, 1 parental variant in BW cross, 0 parental variants in BF crosses, and the singular variant in BW samples shows biallelic expression (53% paternal reads) in epiblast.*
- *Pon2: no parental variants in either cross.*
- *Pon3: not expressed in rat epiblast or EPC.*
- *Pdk4: not expressed in rat epiblast or EPC.*

*These data were pulled from “Additional Data 1 Allele specific RNAseq.xlsx” sheet “rat ratios tests” or directly viewed using this shared UCSC Genome Browser Session that we are happy to share with you:* [*https://genome.ucsc.edu/s/JRA/the_rat_imprintome_share*](https://genome.ucsc.edu/s/JRA/the_rat_imprintome_share)

7.      In addition, how about the situation of many other well-known imprinted gene clusters, such as Peg3, Nnat, Rtl1, Mest and Ndn imprinted genes because many other genes are included in these imprinted clusters. Without such information, it is not possible to compare the mouse and rat genomic imprinting comprehensively.

*Reply: See above. Again, the choice of word “comprehensive” in our manuscript was unintentionally misleading, we have updated the manuscript to instead state that our analyses are “agnostic and systematic”, and that our list of imprinted genes is “extensive” or “unprecedented”. We also indicate that more rat imprinted genes likely exist but were precluded in our experimental design.*

*For the reviewer’s interest:*

- *Peg3: exons do not overlap a parental variant in either cross. We can confirm the Peg3 CGI promoter is maternally methylated DMR in gametes and epiblast and EPCs.*
- *Nnat: Manual inspection shows clear paternal expression of Nnat. Lack of parental variants preclude the identification of the imprinted CGI promoter, though intermediate methylation is observed. Unfortunately, Nnat is moderately expressed and only met our cutoff thresholds in 3/11 replicates and was therefore omitted from our final list of imprinted genes.*
- *Rtl1: not expressed in rat epiblast or EPC.*
- *Ndn: manual inspection reveals paternally biased expression 5/11 replicates in epiblast but neither of our statistical methods (stringent or lenient) called this gene as biased in parental expression.*

8.      Line 334: An additional set of 33 genes that were scored as maternally expressed in rat EPCs were also found to be highly expressed (RPKM>1 from either allele) in adult blood (Sup. fig. 4c-d). Are they rat-specific non-canonical imprinted genes?

*Reply: This point was also raised by Reviewer #1 (comment #1). We clarify that we do not think these are non-canonical imprinted genes, though we cannot exclude the possibility. We include* ***Figure R1*** *above (an old version of Fig. 1d) to illustrate the genomic position of these 33 hits. None of the 33 hits are previously known imprinted genes, though we cannot rule out the fact that these may indeed be maternally expressed imprinted genes in the EPC. We note that, as described in* ***Table R1*** *above, the majority of these genes are associated with blood-related ontologies. We do not count any of these 33 genes in our final list of imprinted genes.*

*The manuscript was updated:*

*Results section: “While an additional set of 33 genes that were scored as maternally expressed only in rat EPCs, we chose not to study these in greater detail because all were found to be highly expressed (RPKM>1 from either allele) in adult blood and thus may reflect maternal decidua expression (Sup. fig. 5c-d, see Sup. table 1).”*

*Discussion section: “Therefore, the full catalogue imprinting in the rat will require additional studies. […] . In addition, extraembryonic samples are frequently contaminated with maternal decidua, making it difficult to discriminate between maternally expressed imprinted genes in our EPC samples from genes that are normally expressed in adult blood.”*

Are they included in the total number of imprinted genes and rat-specific non-canonical imprinted genes or not?

*Reply: As mentioned above in response to Reviewer 1 comment #1, we opt for caution and do not count these 33 hits as candidate imprinted genes, though we do not discount the possibility that any of the 33 maternally expressed imprinted genes in EPC samples could be true imprinted genes. As this manuscript may be used as a resource for future imprinting studies, we chose to present fewer, more convincing candidate imprinted genes in rat.*

If not, why? Is this great difference between mouse and rat a main conclusion of this work?

*Reply: Counting potentially confounding hits as imprinted genes is an unsolved problem in the field (15), as mentioned above. Nevertheless, we now mark the “33 genes that were scored as maternally expressed in rat EPCs that were also found to be highly expressed (RPKM>1 from either allele) in adult blood” as potentially imprinted and provide all salient information about them in* ***Supplemental Table 1 (see Table R1)****.*

9.      Fig. 1c: What are the reads shown in gray in the Igf2/H19 region? Does it mean that only a small part of Igf2 and H19 transcript exhibit paternal and maternal expression?

*Reply: We apologize for the lack of clarity. The gray reads in all our genome browser screenshots showing RNAseq tracks (including* ***Fig. 1c****) represent alignments to the reference genome, i.e. allele-specific information was not considered. Only a portion of any gene can be assessed for parent-of-origin, based on the presence of a parental variant, which we colour red or blue based on originating parental genome. The assumption is that the entire transcript shows consistent parent-of-origin expression. In previous versions of our manuscript, we included the genomic location of parental genetic variants (****see Figure R5****), but we removed these due to space limitations.*

**Figure R5, an alternate version of panel Figure 1c.**

*Reply: Nevertheless, we have updated the figure legends to clarify what the RNAseq track colours represent.*

10.     The authors discussed a variety of mechanism how novel non-canonical imprinted genes emerged. It is better to present them as an additional simple list.

*Reply: As requested, we now include a table (****Table R2 above and Table 2 in the manuscript****) listing rat-specific non-canonical imprinted genes and their salient features (also requested by Reviewer #1, comment #5).*

11.     Concerning DNA methylation of non-canonical imprinted genes. How do the ERVK LTR in Gab1 methylated? Is the upstream promoter of ERVK in Sall1 methylated? Is H3K27me3 not involved in the case of Gag1? What is the mechanical difference between Gab1 and Sall1?

*Reply: These are all good questions. We address them in turn below:*

How do the ERVK LTR in Gab1 methylated?

*Reply: It remains unknown how non-canonical imprints such as the ERVK LTR in Gab1 switch from maternal H3K27me3 to maternal DNA methylation in EPCs. One hypothesis suggests it is caused by promiscuous DNA methylation in the extra-embryo following implantation, with the paternal allele being protected against such de novo DNA methylation activity by H3K4me3 (16).*

Is the upstream promoter of ERVK in Sall1 methylated?

*Reply: Our datasets did not contain sufficient information to agnostically call a parental DMR at the upstream ERVK promoter of Sall1 in EPC samples (see* ***Figure R6****).*

**Figure R6** Paternal-specific expression of *Sall1* is promoted by a seemingly imprinted ERVK element.

*Reply: Of note, a few (but insufficient in number, <4) CpGs are hypermethylated on the maternal allele and hypomethylated on the paternal allele over the alternate ERVK promoter of Sall1 (****Figure R6*** *yellow box, ERVK in red). Consistently, allele-agnostic DNAme levels (which are an average of both alleles and are not subject to loss of information due to a lack of parental polymorphisms) show intermediate DNAme levels over the ERVK element, which may potentially reflect a DMR.*

Is H3K27me3 not involved in the case of Gag1?

*Reply: H3K27me3 is involved in non-canonical imprinting of Gab1, as maternal KO of Eed results in loss of Gab1 imprinted expression (17). However, maternal H3K27me3 is lost following implantation (16, 18), so the mark, per se, cannot be responsible for the maintenance of imprinting. More investigation is required to reveal which epigenetic modification in EPC cells is required for maintenance of non-canonical imprinting.*

What is the mechanical difference between Gab1 and Sall1?

*Reply: Unfortunately, the best study to answer this question (Andergassen et al. 2021, Dev Cell) did not capture Sall1 as an imprinted gene in extra-embryonic cells. Therefore, the loss of imprinted expression of Sall1 was not assessed in zygotic DNMT1, PRC1, PRC2 or G9a-GLP KO embryos (19). Regardless, two independent studies demonstrated that Sall1 imprinting was lost in Eed maternal KO (20) and maternal G9a KO (21), suggesting both H3K27me3 and H3K9me2 in the oocyte are important for Sall1 imprinting establishment.*

*Gab1 was also shown to depend on both pathways in these two independent studies, as well as in Andergassen et al.(19), reinforcing the idea that both pathways are important for the imprinted expression of Gab1. Given the strong synteny at Sall1 and Gab1 between rat and mouse (****Sup. Fig. 9b,d****) and similar oocyte chromatin profiles in both species (****Sup. Fig. 9a,c****), we hypothesize that both genes are under the control of a shared regulatory mechanism, namely non-canonical imprinting, and mention this in the revised Results section.*

*A new sentence was added (p.21): “Thus, in Muridae, both genes are likely governed by an LTR-dependent non-canonical imprinting mechanism.”*

12.     In introduction, the authors introduced that the brown Norway rat is an important model for human pathophysiology and evaluated the utility of the rat in modeling human diseases. In these studies, they clearly demonstrated the difference between rats and humans in terms of no-canonical imprinting. Therefore, this reviewer think that they should comment the applicability and/or limitation of using rat in these studies.

*Reply: This is an important discussion point that we now elaborate on in the revised manuscript. In short, we emphasize the usefulness of the rat as a model of canonical imprinting in the human. However, this is not the case for non-canonical imprinted genes, which are seemingly not conserved between rat and human (and only slightly between rat and mouse for that matter). Indeed, despite the limitations of our experimental design, we nevertheless identify 8 novel imprinted genes in rat, all of which show evidence of non-canonical imprinting regulation. We further discuss that understanding the (potentially) shared molecular function of non-canonical imprinted genes in general, including in alternate model organisms, will deepen our understanding of placental imprinting evolution. The revised text now reads:*

*“Finally, there is an increasing body of evidence implicating canonical imprinted genes in complex behavioural traits (22–27). Our observation that canonical imprinted gene expression is highly conserved in the rat opens the door to future mechanistic studies on the role of these genes in behavioural and other traits in this increasingly tractable model.”*

*Meanwhile, we expand on the usefulness of studying species-specific imprinting (which happens to be non-canonical and exclusive to EPCs) to better understand the evolution of eutherians (below).*

13.     What do authors think about the applicability of the variation of the non-canonical imprinted genes to explain species-specific evolution of other eutherian lineages rather than rodents?

*Reply: This is undoubtedly an important line of future investigation. The revised Discussion now reads:*

*“Furthermore, whether the imprinted expression of rat-specific imprinted genes contribute to differences in rat and mouse physiology and/or behaviour remains to be established. Regardless, as the placenta is a particularly rapidly evolving tissue (80, 81), we speculate that the rapid evolution of non-canonical imprinting may contribute to this process. Indeed, the idea that imprinting drove placental evolution and viviparity was the subject of a recent review (82). Furthermore, ERVK elements, which frequently overlap non-canonical imprints (15, 17), are a class of retroviruses that have been implicated in the rapid evolution of the placenta (83).”*

14.     How do the authors think the difference between rat and mouse contributed to evolution of these species?

*Reply: It is tempting to speculate that the species-specific non-canonical imprinted genes identified here contribute to the speciation of rats and mice. Comparative mouse and rat placenta studies are required for more detailed characterization in the relevant tissue. Subsequent experiments focused on disruption of imprinting of species-specific imprinted genes could then identify any imprinted expression that may confer species-specific placental physiology.*

Is it unique to rodents?

*Reply: We do not believe non-canonical imprinting writ large is unique to the rodent lineage. On the other hand, a recent study profiling H3K27me3 in the developing embryo in mouse, rat, pig, cow and human revealed that only rodents retain maternal H3K27me3 to the blastocyst stage, suggesting non-canonical imprinting by maternal H3K27me3 is not maintained outside the rodent lineage. However, alternative H3K27me3-independent mechanisms for non-canonical imprinting may exist in other species. For example, the human placenta shows an increased number of paternally expressed imprinted genes in the placenta in association with increased DNAme and H3K9me3 on the maternal allele (28). Furthermore, non-canonical imprinting has been reported in an independent study in the crab-eating macaque and human (29). These points are discussed in the revised discussion.*

Minor points:
1.      Fig. 1d: An asterisk on Sfmbt2 should be omitted because it is not imprinted in human.

*Reply: This has been corrected.*

*We note that there is an intronic imprinted CGI in human placenta and other somatic tissues like brain, lund and spleen. However, due to lack of expression data, we removed the asterisk from Sfmbt2.*
2.      Supplemental Figure S1a top: It should be Epi, not EB.

*Reply: This has been corrected.*
3.      Line 68: There is an unnecessary space.

*Reply: This has been corrected.*
4.      Figure 2 d: what are the paternally expressed reads in the Kcnq1 region (outside of the box) in the mouse?

*Reply: There are no paternally expressed reads outside the box in mouse Kcnq1 – in fact, there are only maternally expressed reads. The figure and annotations were updated for clarity.*
5.      Figure 3c, d: why the Zfp64 and LOC108358526 reads in EPC are maternally expressed?
*Reply: The blue refers to paternal-specific read alignments, therefore Zfp64 and LOC108358526 are paternally expressed. As above we’ve updated the figure and annotations to minimize potential confusion.*

*References cited in Response to referees*

*1.	Pastor WA, Kwon SY. Distinctive aspects of the placental epigenome and theories as to how they arise. Cell Mol Life Sci. 2022;79:569.*

*2.	Hamada H, Okae H, Toh H et al. Allele-Specific Methylome and Transcriptome Analysis Reveals Widespread Imprinting in the Human Placenta. The American Journal of Human Genetics. 2016;99:1045-1058.*

*3.	Miri K, Latham K, Panning B, Zhong Z, Andersen A, Varmuza S. The imprinted polycomb group gene Sfmbt2 is required for trophoblast maintenance and placenta development. Development. 2013;140:4480-4489.*

*4.	Itoh M, Yoshida Y, Nishida K, Narimatsu M, Hibi M, Hirano T. Role of Gab1 in heart, placenta, and skin development and growth factor- and cytokine-induced extracellular signal-regulated kinase mitogen-activated protein kinase activation. Mol Cell Biol. 2000;20:3695-3704.*

*5.	Kobayashi H. Canonical and Non-canonical Genomic Imprinting in Rodents. Frontiers in Cell and Developmental Biology. 2021;9*

*6.	Chen Z, Hagen DE, Wang J et al. Global assessment of imprinted gene expression in the bovine conceptus by next generation sequencing. Epigenetics. 2016;11:501-516.*

*7.	Dong Y, Jin L, Liu X et al. IMPACT and OSBPL1A are two isoform-specific imprinted genes in bovines. Theriogenology. 2022;184:100-109.*

*8.	Bischoff SR, Tsai S, Hardison N et al. Characterization of conserved and nonconserved imprinted genes in swine. Biol Reprod. 2009;81:906-920.*

*9.	Ding YY, Liu LY, Zhou J et al. Detection of a novel single nucleotide polymorphism and imprinted status analysis of the Ras protein-specific guanine nucleotide-releasing factor 1 gene in domestic pigs. Genet Mol Res. 2014;13:10574-10581.*

*10.	Oczkowicz M, Szmatoła T, Piórkowska K, Ropka-Molik K. Variant calling from RNA-seq data of the brain transcriptome of pigs and its application for allele-specific expression and imprinting analysis. Gene. 2018;641:367-375.*

*11.	Ahn J, Wu H, Lee J et al. Identification of a Novel Imprinted Transcript in the Porcine GNAS Complex Locus Using Methylome and Transcriptome of Parthenogenetic Fetuses. Genes (Basel). 2020;11:E96.*

*12.	Suzuki S, Renfree MB, Pask AJ et al. Genomic imprinting of IGF2, p57(KIP2) and PEG1/MEST in a marsupial, the tammar wallaby. Mech Dev. 2005;122:213-222.*

*13.	Suzuki S, Ono R, Narita T et al. Retrotransposon silencing by DNA methylation can drive mammalian genomic imprinting. PLoS Genet. 2007;3:e55.*

*14.	Ishihara T, Griffith OW, Suzuki S, Renfree MB. Placental imprinting of SLC22A3 in the IGF2R imprinted domain is conserved in therian mammals. Epigenetics Chromatin. 2022;15:32.*

*15.	Okae H, Hiura H, Nishida Y et al. Re-investigation and RNA sequencing-based identification of genes with placenta-specific imprinted expression. Human Molecular Genetics. 2012;21:548-558.*

*16.	Chen Z, Yin Q, Inoue A, Zhang C, Zhang Y. Allelic H3K27me3 to allelic DNA methylation switch maintains noncanonical imprinting in extraembryonic cells. Sci Adv. 2019;5:eaay7246.*

*17.	Inoue A, Chen Z, Yin Q, Zhang Y. Maternal Eed knockout causes loss of H3K27me3 imprinting and random X inactivation in the extraembryonic cells. Genes Dev. 2018;32:1525-1536.*

*18.	Hanna CW, Pérez-Palacios R, Gahurova L et al. Endogenous retroviral insertions drive non- canonical imprinting in extra-embryonic tissues. Genome Biology. 2019;20:1-17.*

*19.	Andergassen D, Smith ZD, Kretzmer H, Rinn JL, Meissner A. Diverse epigenetic mechanisms maintain parental imprints within the embryonic and extraembryonic lineages. Dev Cell. 2021;56:2995-3005.e4.*

*20.	Santini L, Halbritter F, Titz-Teixeira F et al. Genomic imprinting in mouse blastocysts is predominantly associated with H3K27me3. Nat Commun. 2021;12:3804.*

*21.	Zeng TB, Pierce N, Liao J, Szabó PE. H3K9 methyltransferase EHMT2/G9a controls ERVK-driven noncanonical imprinted genes. Epigenomics. 2021;13:1299-1314.*

*22.	Lefebvre L, Viville S, Barton SC, Ishino F, Keverne EB, Surani MA. Abnormal maternal behaviour and growth retardation associated with loss of the imprinted gene Mest. Nature genetics. 1998;20:163-169.*

*23.	Li L, Keverne EB, Aparicio SA, Ishino F, Barton SC, Surani MA. Regulation of maternal behavior and offspring growth by paternally expressed Peg3. Science. 1999;284:330-333.*

*24.	Horsthemke B, Wagstaff J. Mechanisms of imprinting of the Prader-Willi/Angelman region. American journal of medical genetics Part A. 2008;146A:2041-2052.*

*25.	Tucci V, Isles AR, Kelsey G et al. Genomic imprinting and physiological processes in mammals. Cell. 2019;176:952-965.*

*26.	Glaser J, Iranzo J, Borensztein M et al. The imprinted Zdbf2 gene finely tunes control of feeding and growth in neonates. eLife. 2022;11*

*27.	Bonthuis PJ, Steinwand S, Stacher Hörndli CN et al. Noncanonical genomic imprinting in the monoamine system determines naturalistic foraging and brain-adrenal axis functions. Cell Rep. 2022;38:110500.*

*28.	Monteagudo-Sánchez A, Sánchez-Delgado M, Mora JRH et al. Differences in expression rather than methylation at placenta-specific imprinted loci is associated with intrauterine growth restriction. Clinical Epigenetics. 2019;11*

*29.	Chu C, Zhang W, Kang Y et al. Analysis of developmental imprinting dynamics in primates using SNP-free methods to identify imprinting defects in cloned placenta. Dev Cell. 2021S1534-5807(21)00730.*

**Second round of review**

**Reviewer 1**

I appreciate the efforts the authors have made to address the issued I raised as well as those raised by the second reviewer. In particular, the authors make important qualifications to their study and analyses; this includes the following critical points:

1. the caveat that they do not provide a comprehensive survey of imprinting in the rat (text changed in various places, including the abstract), because of the the relative sparsity of informative SNPs and incomplete nature of the rat genome;

2. the explanation for not further analysing the 33 genes with maternal allele biased expression in rat EPC, because of the possibility of maternal cell contamination, which could lead to spurious imprinting calls;

3. the addition of two summary tables that helpfully bring the information about the canonical and non-canonical imprinted genes into a single place, including the comparisons with other species including mouse and human.

With these amendments to the manuscript, I consider the authors have satisfactorily resolved the issues I raised.

**Reviewer 2**

The revised version of manuscript has been improved very much and now it becomes easy to understand by additional two Tables. This reviewer is satisfied with all the authors' responses and their discussions in this revised version of manuscript.

Minor comment.

Line 53, first sentence of Introduction: no verb in this sentence.
